# Techniques for the Application of Stereotactic Head Frames Based on a 25-Year Experience

**DOI:** 10.7759/cureus.543

**Published:** 2016-03-25

**Authors:** Michael Safaee, John Burke, Michael W. McDermott

**Affiliations:** 1 Department of Neurological Surgery, University of California, San Francisco

**Keywords:** head frame, stereotactic, gamma knife

## Abstract

The use of skull fixed stereotactic head frames remains an integral part of neurosurgical practice. Methods for the positioning, anesthesia, and fixation have been described in various publications. The authors describe the steps used currently that reflect a 25-year experience with stereotactic frame application. Photographs were obtained throughout the set-up and frame application process. The step-by-step methods were described with accompanying figures. Consent was obtained from that patient to allow for photographs throughout the frame application process. Consent was also obtained from a separate patient for videotaping the entire application process. Descriptive tags are embedded in the video to assist with the instruction of the senior author's (MWM) methods. The senior author has used the described method in over 1,000 cases. A recent analysis of the patient pain experience has been reported and is well tolerated. Supplemental devices beyond the manufacturers' standard equipment have been employed or developed: ethyl chloride spray, angled front posts, frame positioner, and torque wrenches. There have been no shunt perforations, no cranial vault penetrations, one titanium mesh cranioplasty deformation, three pin site infections (3 patients; 4,000 pin sites; 0.075%), and one thermal injury (0.025%). Stereotactic head frame application remains an important part of neurosurgical practice. The steps in application employed here after a 25-year experience appear to make the procedure well tolerated by patients. The authors hope this instructional article will be of value to new users who practice stereotactic radiosurgery, frame-based biopsy, depth electrode placement, or deep brain stimulator implantation.

## Introduction

The use of stereotactic head frames remains an integral part of neurosurgical practice today for the implantation of deep brain stimulators, depth electrodes, stereotactic brain biopsy, and cranial radiosurgery. Unfortunately, instructional methods for the safe, correct, and pain-sparing application are lacking. The advent of image-guided surgical systems for brain biopsy and radiosurgery, in particular, has resulted in less exposure to frame application methods for residents in training. We previously reported on the development of a frame positioning device and angled front bars for the Leksell (Elekta, Atlanta, GA, USA) and CRW (Integra, Plainsboro, NJ, USA) frame systems and have reported on the patient pain experience with head frame application [1-2].

This paper and an online video were created to provide enduring materials that residents may reference for their own training and practice, and to allow patients to see the steps in the procedure prior to the day of treatment. The American Board of Neurological Surgeons now requires the application of stereotactic frames for completion of neurosurgical residency in the United States, making this resource particularly timely. A preoperative checklist, the materials used, and steps in the procedure are documented with still images and an online video.

Photographs were obtained throughout the set-up and frame application process. The step-by-step methods were described with accompanying figures. Consent was obtained from the patient to allow for photographs throughout the frame application process. Consent was also obtained from a separate patient for videotaping the entire application process and descriptive tags embedded in the video to assist with the instruction of the senior authors methods.

## Technical report

The senior author has developed the technique described herein over a 25-year period, and a prior report has documented the patient pain experience with head frame application using these methods [2]. Done correctly, frame application under local anesthesia with mild intravenous sedation can be accomplished and appears to be well-tolerated.

Prior to frame application, the surgeon should review details of the patient's prior neurosurgical history and confirm these verbally with the patient during the pre-procedure time-out (Table 1). Once the checklist has been reviewed, any available imaging should be reviewed to check for shunt hardware, cranial defects, or cranioplasty materials. Knowing where shunt catheters and valves may be located over the parietal occipital region will allow the surgeon to identify these by palpation on the patient and adjust frame angles of inclination, rotation, and rear bar length. The positions of these materials can be marked on the skin for avoidance. Frontal, parietal, and occipital craniectomy, craniotomy, or cranioplasty sites may also require modifications of frame positioning and screw tightening.

**Table 1:** Frame Application Checklist

Patient review
☐ Prior shunt
☐ Side of shunt and location of catheter
☐ Prior craniotomy
☐ Date of craniotomy
☐ Side of craniotomy (left/right/midline)
☐ Type of craniotomy (frontal/parietal/occipital/pterional)
☐ History of local anesthesia experience
☐ Need for more than normal amounts
☐ Medication allergies
Imaging review
☐ Plain XR
☐ CT head/brain
☐ MRI head/brain
Equipment review
☐ IV access
☐ Frame components complete
☐ Pin and post sets available
☐ Topical and local anesthesia available
☐ Torque wrenches available

**Figure 1:**
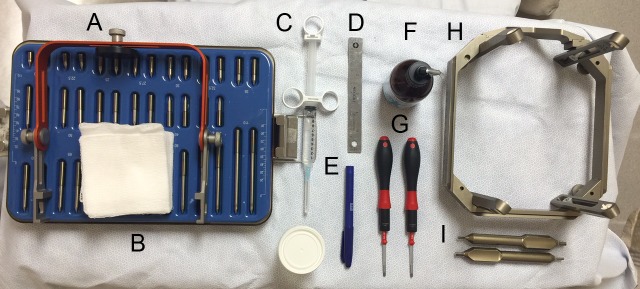
Equipment for frame application. Frame positioner (A), frame pin/screw set (B), local anesthetic (C), ruler (D), marking pen (E), ethyl chloride spray (F), torque wrenches (G), Leksell frame (H), pin/screw wrenches (I).

**Figure 2:**
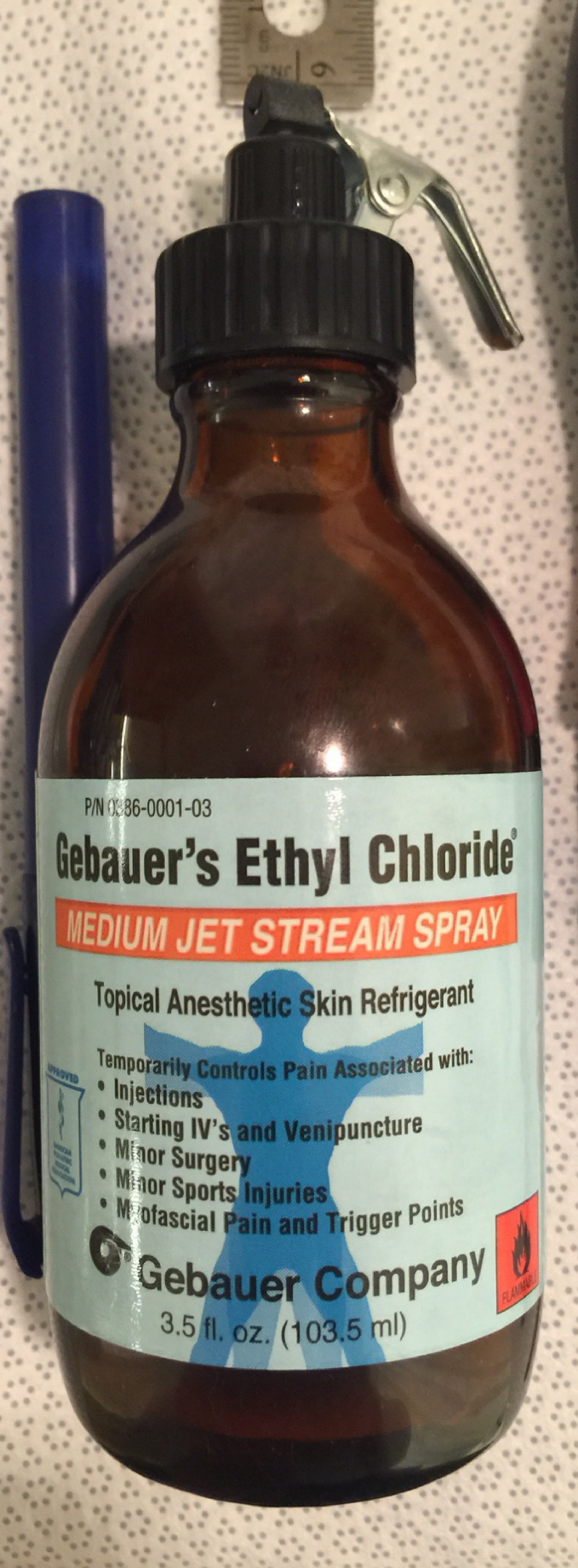
Gebauer’s ethyl chloride® instant topical anesthetic (Gebauer Company, Cleveland, OH, USA).

A standard frame placement tray includes a frame positioner (Q-Surgical, Sausalito, CA, USA), frame pin/screw set, local anesthetic (Xylocaine/Marcaine), ruler, marking pen, ethyl chloride spray (Gebauer Company, Cleveland, OH, USA), torque wrenches (Wiha Tools, Monticello, MN, USA), Leksell frame, and pin/screw wrenches (Figure 1). The ethyl chloride spray quickly anesthetizes the skin and is acquired from the Gebauer Company (Figure 2).

**Figure 3:**
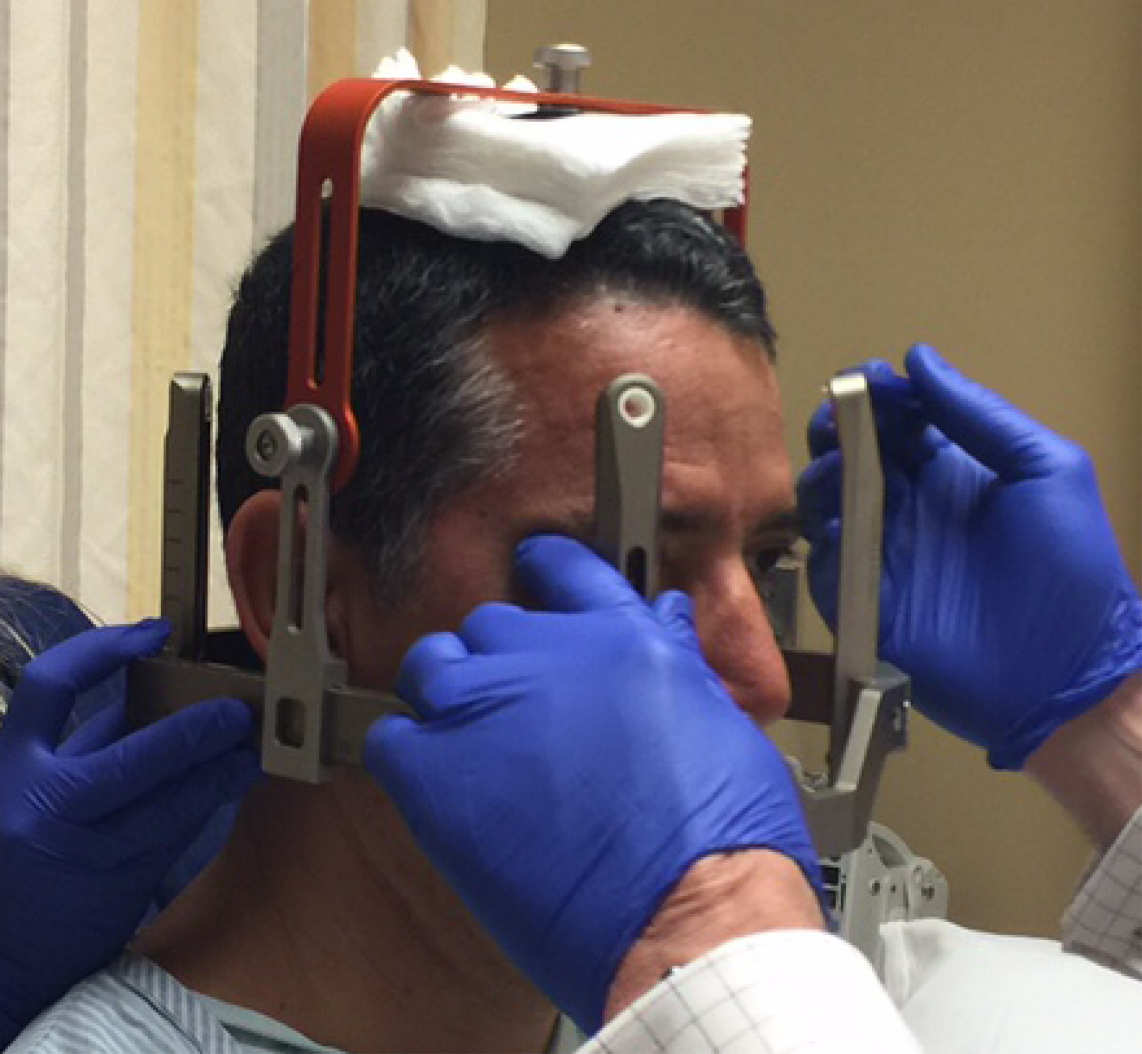
The frame holder is positioned on top of the patient’s head with gauze padding for initial frame positioning.

**Figure 4:**
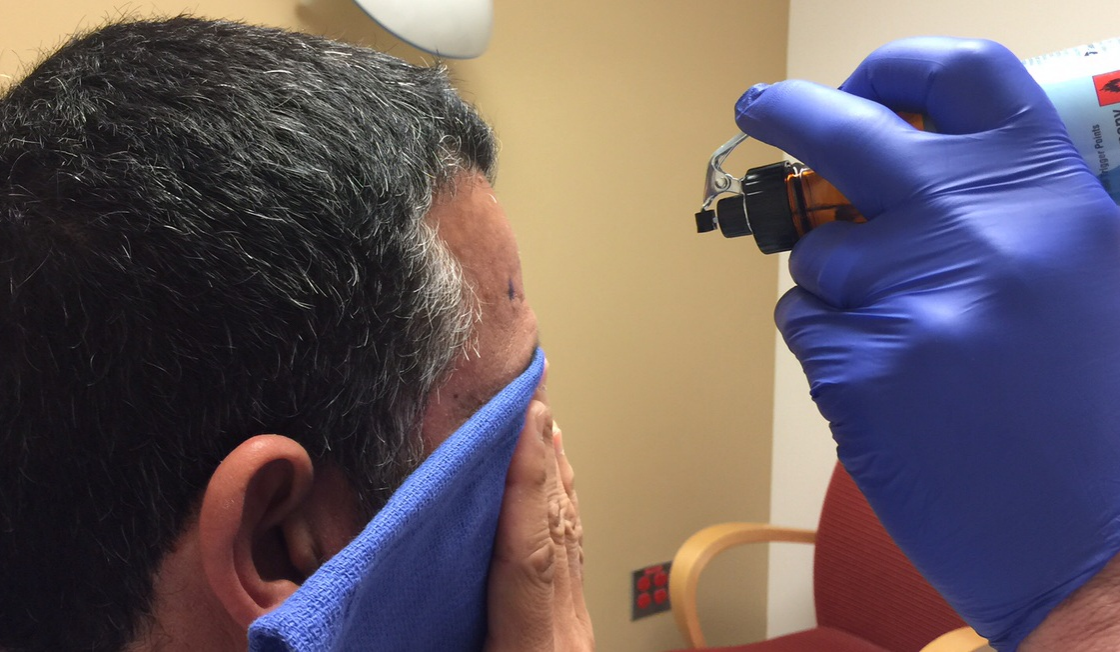
Injection sites are sprayed with ethyl chloride to anesthetize the skin.

**Figure 5:**
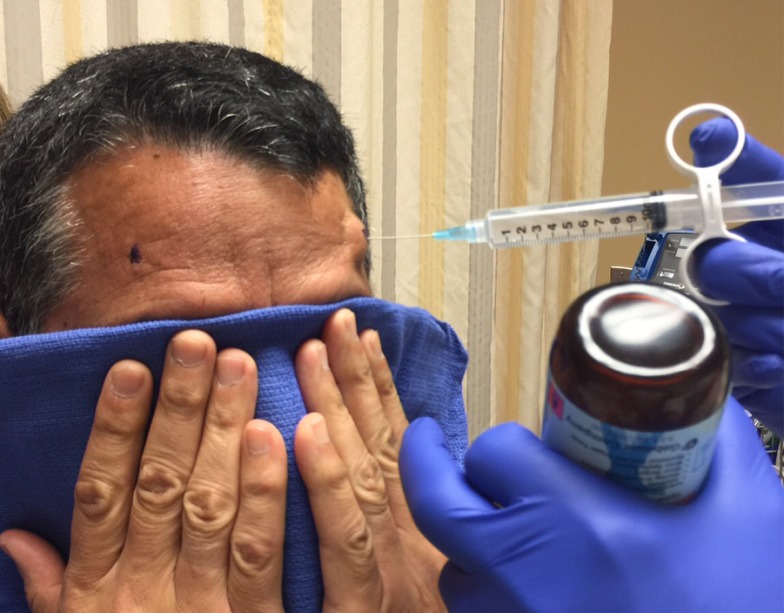
Each pin site is injected with 2 ml of local anesthetic.

**Figure 6:**
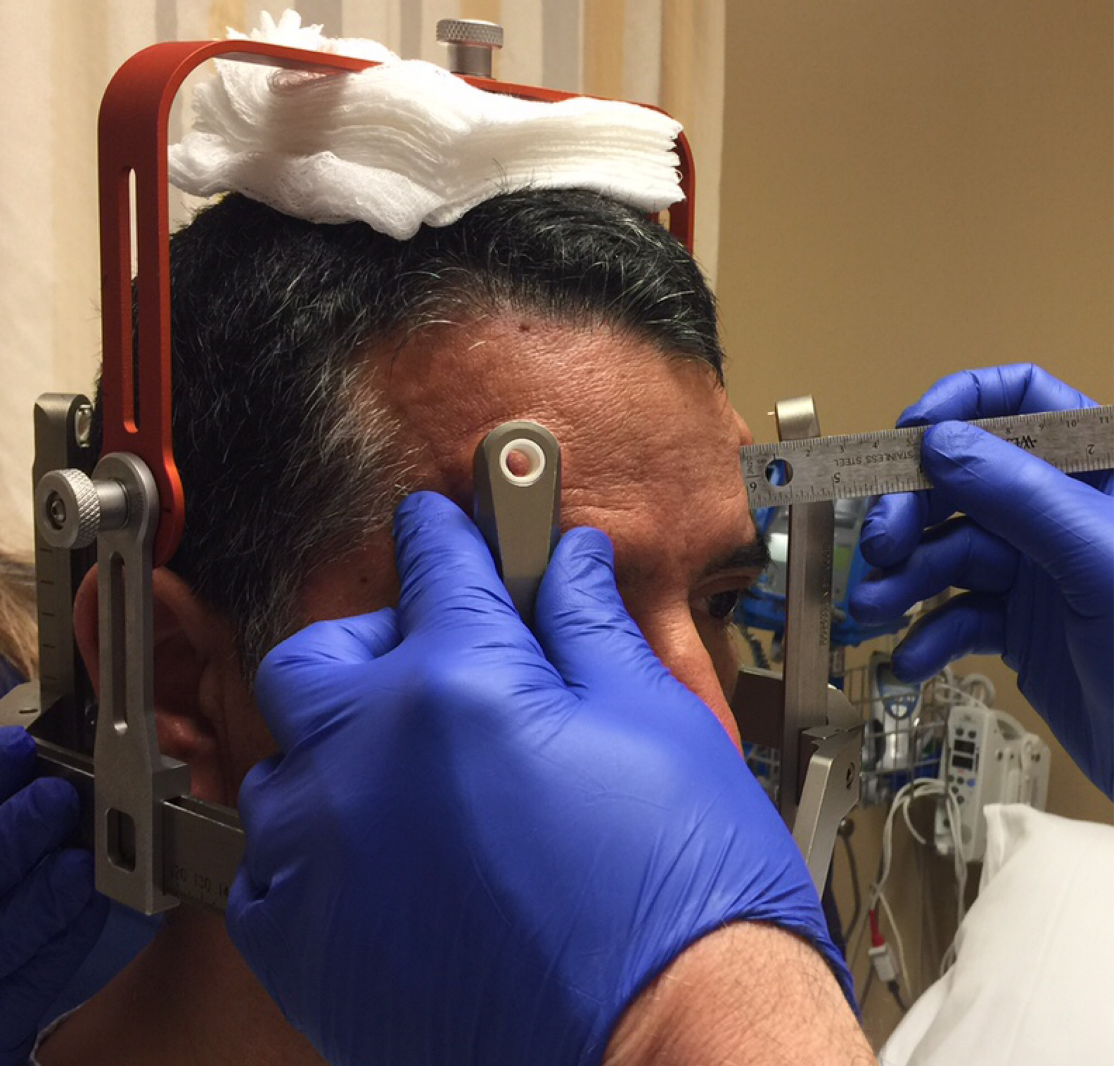
Distance from skin to outer edge of post is measured to estimate pin/screw length.

**Figure 7:**
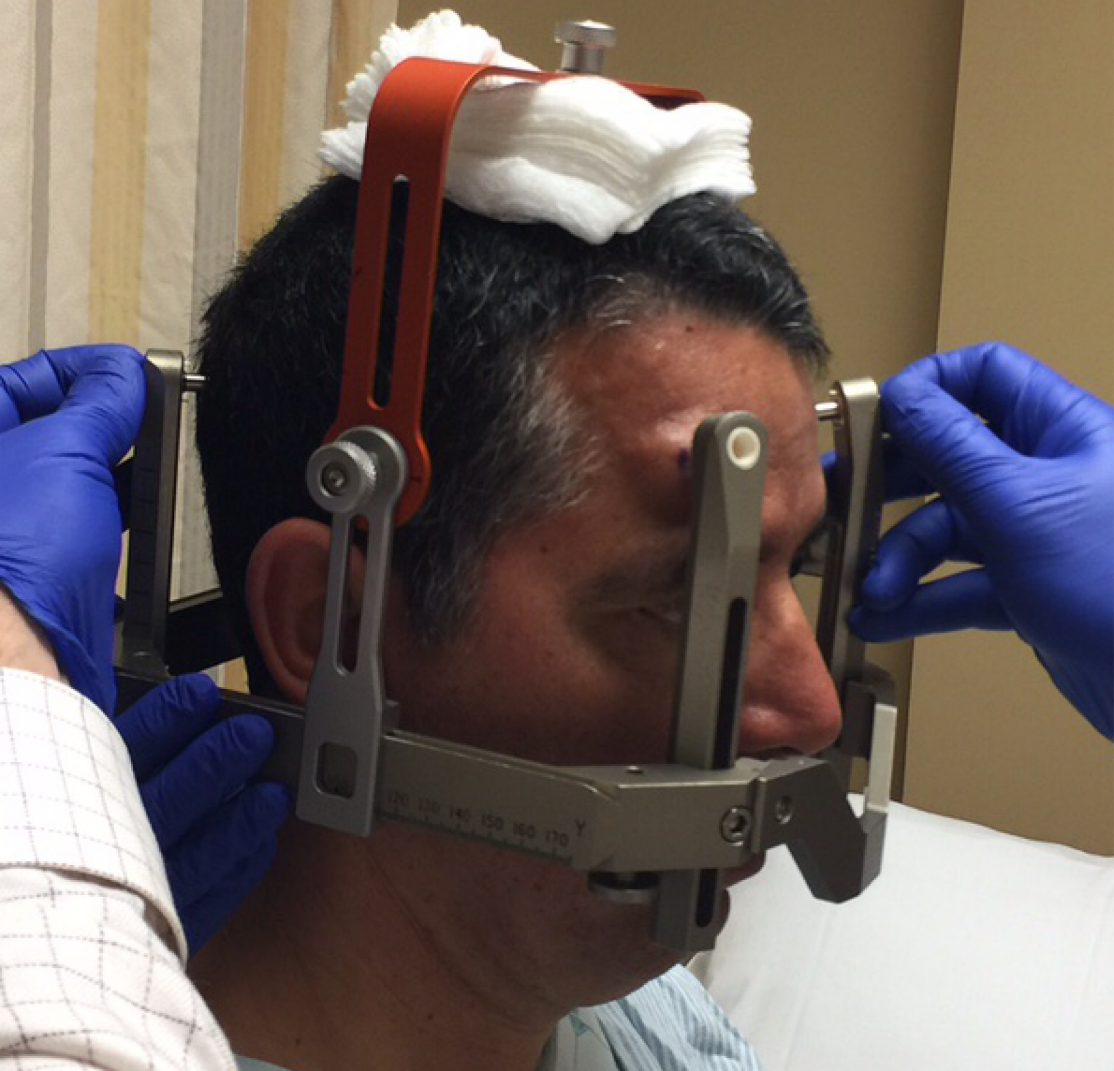
Pins are manually placed on the diagonal.

**Figure 8:**
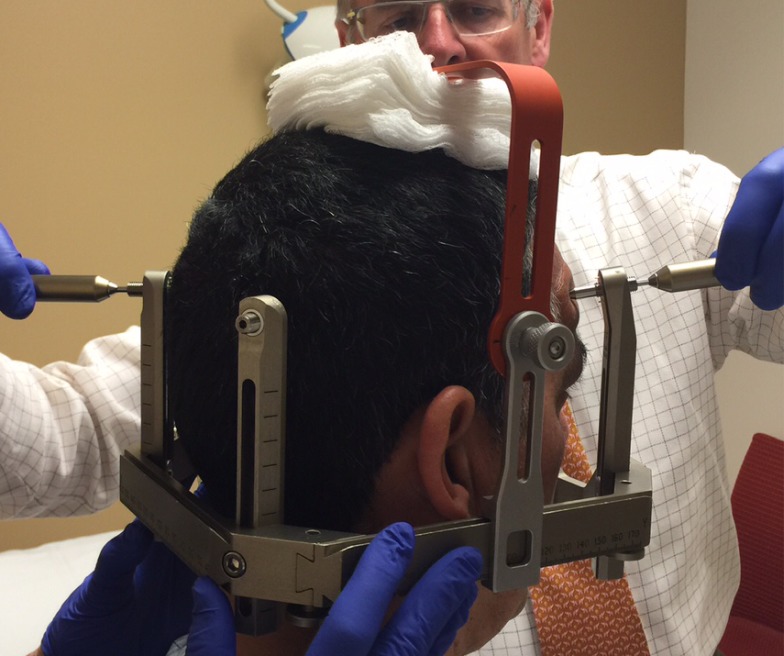
Pins are tightened on the diagonal with thumb and index finger grip.

**Figure 9:**
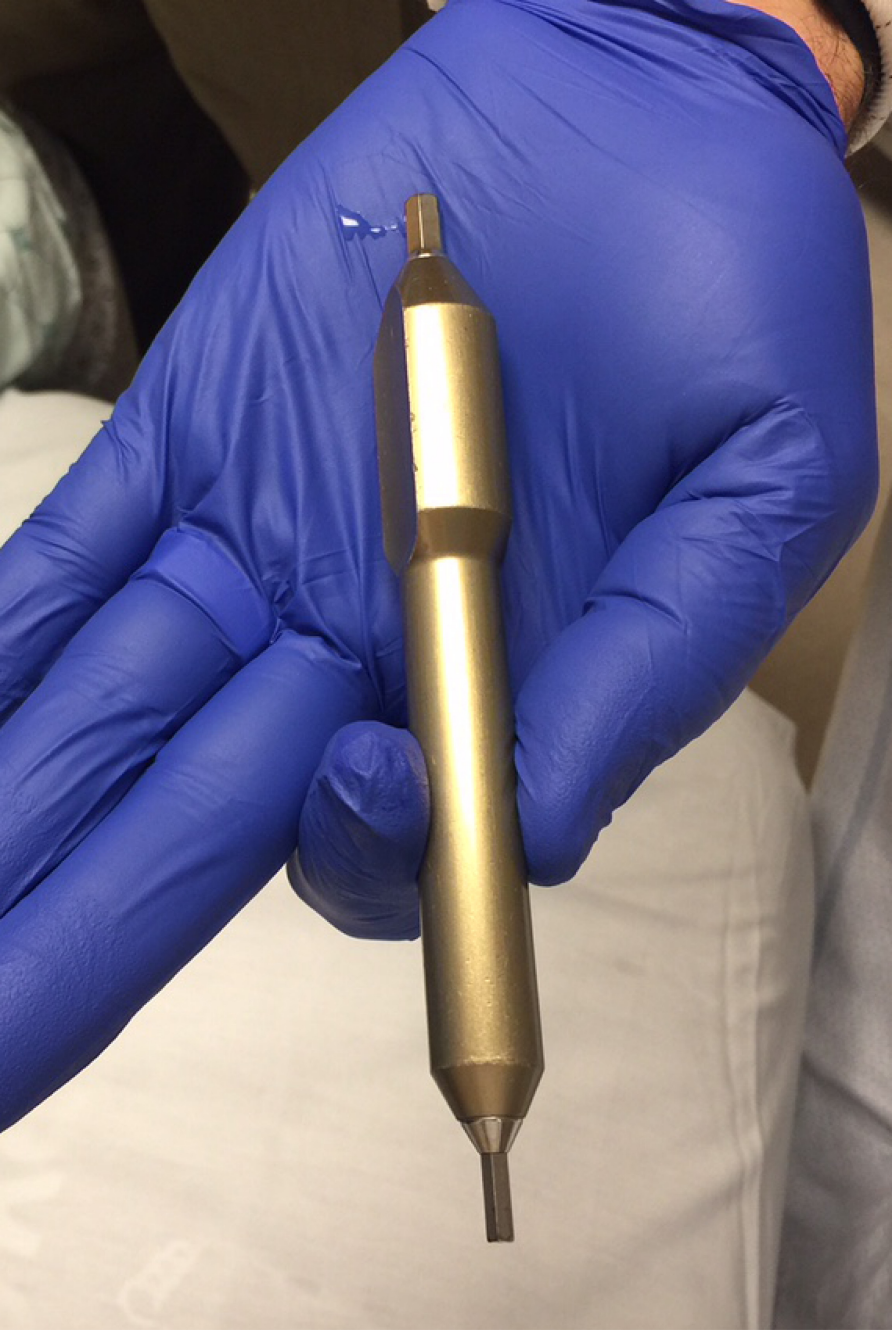
The wrench is held with the thumb and index finger to avoid over-tightening the screws prior to the final torque.

**Figure 10:**
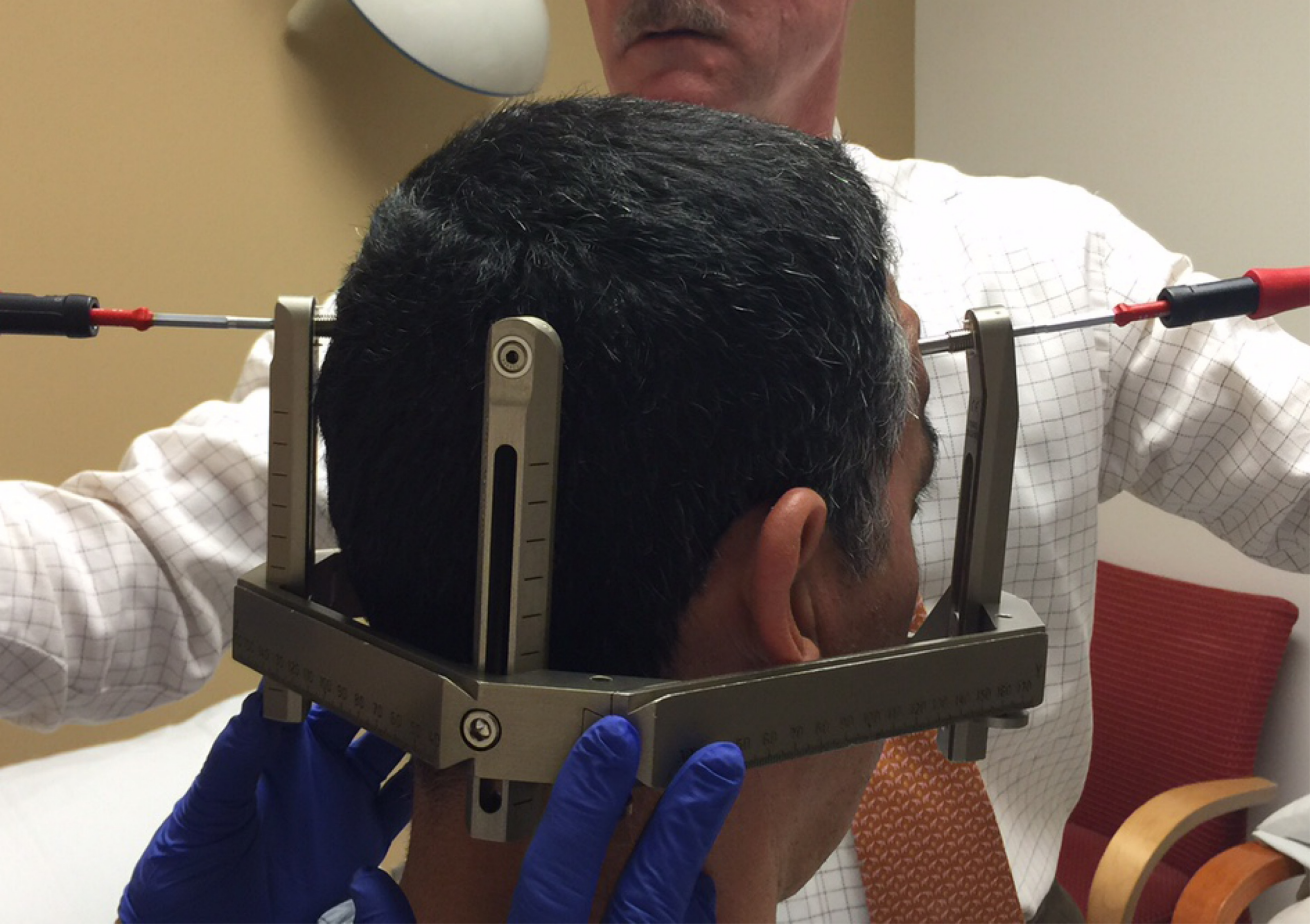
Final screw tightening is performed with torque wrenches on the diagonal to 4 inch-pounds of torque per screw.

Before the application begins, the patient has the option of receiving some mild intravenous sedation, usually lorazepam, 0.5 – 1.0 mg. A pictorial representation of the frame application is provided. The frame is positioned with the patient sitting up facing the surgeon using cotton gauzes as spaces to adjust the vertical height of the frame (Figure 3). The projected pin/screw entry sites and the superior temporal line are marked with a marking pen and then the frame is removed. Care is taken to make sure the frontal pins do not penetrate the skin behind the superior temporal line and angled front bars are used, if necessary [2]. The patient’s eyes are covered with a blue towel and ethyl chloride is sprayed onto a marked pin site (Figure 4), followed immediately by the injection of a local anesthetic (Figure 5). This process is repeated for each of the four-pin/screw sites. The frame is then repositioned, and the distance between the skin and the outer aspect of the bar at each pin/screw site is made (Figure 6). In general, 5-8 mm is added to this measurement to account for scalp thickness, which varies depending on body habitus, and then the pins/screws are inserted on opposite sites and tightened by finger (Figure 7). After all four pins are in place, the pin wrenches are used to tighten the pins on the diagonal (Figure 8). Tightening is performed using the thumb and index finger for grasping so as to limit the amount of torque that can be applied (Figure 9). We use custom torque wrenches to tighten to pins/screws to 4 inch-pounds so as to secure the frame adequately and lessen frame bending and distortion (Figure 10). Once the click is heard from the torque wrenches, screw tightening is complete and the frame holder is removed. The patient can now be moved to radiology for imaging. The complete procedure has been documented and can be viewed at https://youtu.be/z0ZJ9sDhST8.

**Figure 11:**
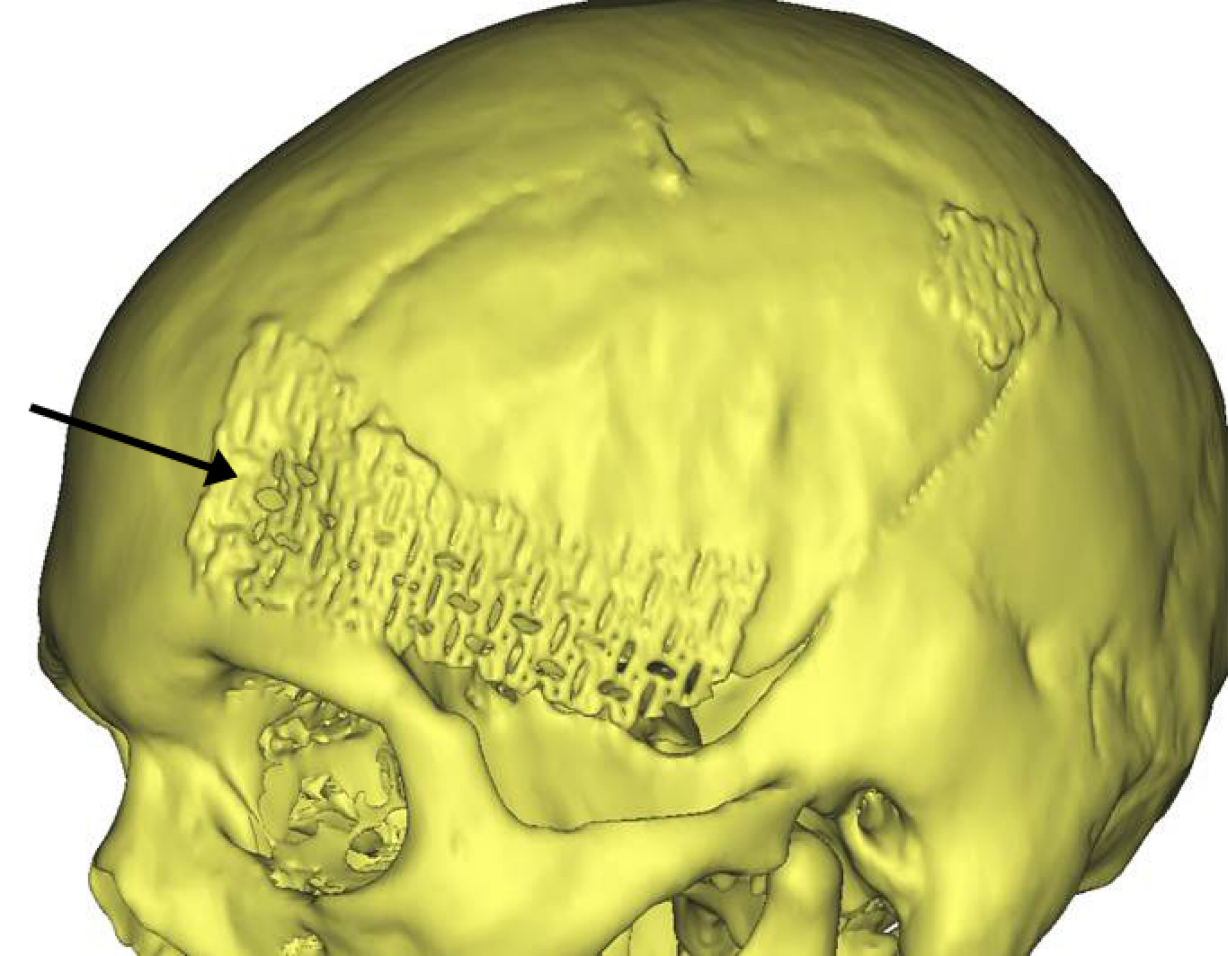
Deformation of titanium mesh cranioplasty caused by pin application. This injury caused a cosmetic deformity that required revision cranioplasty.

There have been no shunt perforations, cranial vault penetrations, one titanium mesh cranioplasty deformation, three pin site infections (3 patients; 4,000 pin sites; 0.075%), and one thermal injury (0.025%). In one patient who had a pterional craniotomy for an aneurysm, the senior author neglected to check the postoperative CT scan for the extent of the cranioplasty that was done using titanium mesh alone, which is relatively weak, causing an indentation of the mesh that resulted in a cosmetic deformity (Figure 11) [3-6]. Because of the indentation created over the non-hear-bearing scalp, a revision cranioplasty was performed using a custom implant.

## Discussion

Currently, in the United States, there are 102 residency training programs with an entire generation of residents who must be well-versed in the proper application of stereotactic head frame placement. The American Board of Neurological Surgery (ABNS) requires that residents perform at least 10 radiosurgery cases, 10 functional procedures, and five stereotactic frame placements. Potentially, this would mean that 20 frame-based procedures could be done using a variety of techniques with a variety of patient experiences. This number may be an overestimate since both radiosurgery and deep brain stimulation electrode placement can be done with non-frame-based image-guided methods. However, at our first-year resident training boot camps, stereotactic frame placement is not currently part of the curriculum; therefore, it was felt that documentation of this method of application would provide an enduring material reference for residents currently in training.

Our institutional practice is to utilize angled front posts to allow for pin contacts with the cranium anterior to the superior temporal line, despite the lateral or posterior position of the frame. At that time, our experience in 536 cases (90 vascular lesions, 446 tumors) found that the use of these posts reduced the number of times the frame had to be repositioned; this allowed the frame to be positioned as low as possible, which allowed for easier application and provided increased stability. We have also reported on patients’ pain experience with stereotactic frame positioning using this method in a prospective patient cohort [2]. In this study, pain assessments were collected prior to the frame placement, immediately after the frame placement, immediately before and after radiosurgery, and one day after radiosurgery. A standard 0-10 point scale was used, with 0 indicating no pain and 10 indicating worst pain of life. Frame placement was not perceived as a painful procedure with the post-frame placement scores averaging 0.5 - 0.9, with an increase in pain perception after radiosurgery ranging from 1.6 - 2.3 [2]. There were no significant differences when comparing pain perception with the use of straight versus angled frame posts. Given the relatively low pain scores, it is important to counsel patients on frame placement and expected pain experience.

Despite the ubiquitous use of stereotactic frames in neurosurgery, there is a paucity of literature describing techniques for proper frame placement. Kamiryo and Laws described an adjustable applicator for Leksell frame placement [7]. Their applicator consists of four transparent acrylic plates with an inflatable air cuff beneath the top plate and on each side to support the frame at the desired height and permit real-time adjustments to optimize frame position and alignment. This applicator allows for accurate midline frame placement or shifted frame placement when using the adjustable air cuffs. Nazakawa, et al. describe an alternate technique for frame placement using a base plate to support the patient’s head and stereotactic frame [8]. The system consists of transparent acrylic boards with holes that allow for injection of local anesthetic and manipulation of fixation screws. This plate supports the base of the head with an air cuff, which can be inflated or deflated to allow for proper position. The authors have used the system in more than 1,500 patients with good results.

Although frameless techniques are increasing in availability and accuracy, frame-based approaches continue to play an important role. In a study comparing the use of frame-based and non-frame-based techniques for intracranial biopsies, both were equally effective at providing a tissue diagnosis with minimum morbidity and mortality; however, frame-based approaches were shown to require fewer anesthesia resources and produce shorter operating room times and hospital stays [9]. Given their critical role in modern neurosurgery, minimal pain and morbidity, and a high degree of accuracy, proper frame application remains a critical technique for neurosurgeons in training.

## Conclusions

Stereotactic head frame application remains an integral part of neurosurgical practice. We present a frame application employed across a 25-year experience that makes the procedure well tolerated by patients. The authors hope this instructional article will be of value to both new and experienced users who practice stereotactic radiosurgery, frame-based biopsy, depth electrode placement, and deep brain stimulator implantation.
